# Supplementary material for: Impact of pyrazinamide usage on serious adverse events in elderly tuberculosis patients: A multicenter cohort study
Source: PLoS One. 2024 Sep 26;19(9):e0309902. doi: 10.1371/journal.pone.0309902 (PMC11426458; doi:10.1371/journal.pone.0309902)
Supplement: S1 Table — (DOCX) [file pone.0309902.s001.docx]

Supporting Table 1. Baseline characteristics and treatment results based on pyrazinamide usage in patients aged 75 years and older

| Variables | Total, n = 199 | Without PZA,  n = 26 (13.1%) | With PZA,  n = 173 (86.9%) | *P* value |
| --- | --- | --- | --- | --- |
| Baseline characters | | | | |
| Age, years | 81.62 ± 4.64 | 82.54 ± 4.58 | 81.14 ± 4.61 | 0.150 |
| Males, n (%) | 89 (44.7) | 10 (38.5) | 79 (45.7) | 0.491 |
| BMI, kg/m^2^ | 21.84 ± 3.61 | 22.61 ± 4.37 | 21.74 ± 3.37 | 0.366 |
| Ever smoker (%) | 62 (31.2) | 8 (30.8) | 54 (31.2) | 0.964 |
| Re-treatment. n/N (%) | 28/197 (14.2) | 4/25 (16.0) | 24/172 (14.0) | 0.784 |
| Extrapulmonary TB, n (%) | 24 (12.1) | 5 (19.2) | 19 (11.0) | 0.229 |
| Multi-lobe infiltration, n/N (%) | 183/198 (92.4) | 26/26 (100.0) | 157/172 (91.3) | 0.117 |
| Cavitary lesion, n/N (%) | 17/196 (8.7) | 1/26 (3.8) | 16/170 (9.4) | 0.348 |
| AFB Smear positive, n/N (%) | 111/167 (66.5) | 12/18 (66.7) | 99/149 (66.4) | 0.985 |
| Chronic pulmonary disease, n (%) | 22 (11.1) | 6 (23.1) | 16 (9.2) | 0.036 |
| Renal disease, n (%) | 16 (8.0) | 4 (15.4) | 12 (6.9) | 0.140 |
| Liver disease, n (%) | 3 (1.5) | 1 (3.8) | 2 (1.2) | 0.344 |
| Cancer, n (%) | 21 (10.6) | 3 (11.5) | 18 (10.4) | 0.861 |
| Hemoglobin, g/dL^a^ | 11.57 ± 1.52 | 11.45 ± 1.62 | 11.53 ± 1.51 | 0.802 |
| Albumin, g/dL^b^ | 3.57 ± 0.63 | 3.15 ± 0.71 | 3.62 ± 0.59 | 0.001 |
| Total bilirubin, mg/dL ^c^ | 0.65 ± 0.61 | 1.11 ± 1.36 | 0.58 ± 0.29 | 0.069 |
| AST, IU/L ^d^ | 20.26 ± 20.52 | 46.08 ± 55.71 | 27.78 ± 23.51 | 0.125 |
| ALT, IU/L ^e^ | 20.26 ± 20.52 | 24.13 ± 27.61 | 18.94 ± 18.16 | 0.230 |
| Creatinine(mg/dL) ^f^ | 0.96 ± 0.50 | 1.09 ± 0.52 | 0.93 ± 0.48 | 0.147 |
| Treatment Results | | | | |
| Treatment Success, n (%) | 165 (82.9) | 16 (61.5) | 149 (86.1) | *0.002* |
| SAEs, n (%) | 62 (31.2) | 11 (42.3) | 51 (29.5) | 0.188 |
| Time to first SAE, median days (IQR) | 35 (13-89) | 28 (13-63) | 46 (13-91) | 0.693 |
| Treatment duration, median days (IQR) | 185 (179-217) | 206 (20-180) | 185 (180-203) | 0.273 |
| Medication interruption, n (%) | 20 (10.1) | 2 (7.7) | 18 (10.4) | *0.668* |
| LTFU, n (%) | 5 (2.5) | 2 (7.7) | 3 (1.7) | 0.128 |

Abbreviations: AFB: Acid-Fast Bacillus; ALT: alanine aminotransferase; AST: aspartate aminotransferase; BMI: Body mass index; IQR: interquartile range; LFTU: lost to follow-up; PZA: pyrazinamide; SAE: serious adverse event; TB: Tuberculosis

^a^ Total n = 182; without PZA n = 24; with PZA n = 158

^b^ Total n = 178; without PZA n = 24; with PZA n = 154

^c^ Total n = 179; without PZA n = 24; with PZA n = 155

^d^ Total n = 181; without PZA n = 24; with PZA n = 157

^e^ Total n = 181; without PZA n = 24; with PZA n = 157

^f^ Total n = 181; without PZA n = 24; with PZA n = 157
