# Supplementary material for: Impact of pyrazinamide usage on serious adverse events in elderly tuberculosis patients: A multicenter cohort study
Source: PLoS One. 2024 Sep 26;19(9):e0309902. doi: 10.1371/journal.pone.0309902 (PMC11426458; doi:10.1371/journal.pone.0309902)
Supplement: S2 Table — (DOCX) [file pone.0309902.s002.docx]

Supporting Table 2. Treatment success and primary outcomes based on age

| Variables | Total  n = 921 | Young  n = 531 (57.7%) | Elderly  n = 390 (42.3%) | *P* value |
| --- | --- | --- | --- | --- |
| Treatment Success, n (%) | 857 (93.1) | 515 (97) | 342 (87.7) | *<0.001* |
| PZA use time, median days (IQR) | 62 (56-69) | 62 (56-70) | 61 (55-66) | *0.004* |
| Treatment duration, median days (IQR) | 189 (182-266) | 189 (182-270) | 190 (182-254) | 0.314 |
| SAEs, n (%) | 158 (17.2) | 60 (11.3) | 98 (25.1) | <0.001 |
| Time to first SAE, median days (IQR) | 37 (14-85) | 40 (17-91) | 31 (14-74) | 0.486 |
| SAE Category |  |  |  |  |
| Hepatotoxicity, n (%) | 47 (5.1) | 17 (3.2) | 30 (7.7) | <0.002 |
| Generalized weakness, n (%) | 19 (2.1) | 2 (0.4) | 17 (4.4) | <0.001 |
| Cytopenia, n (%) | 23 (2.5) | 11 (2.1) | 12 (3.1) | 0.334 |
| Infection, n (%) | 14 (1.5) | 2 (0.4) | 12 (3.1) | 0.001 |
| Dyspnea, n (%) | 13 (1.4) | 2 (0.4) | 11 (2.8) | 0.002 |
| Gastrointestinal adverse drug reactions, n (%) | 14 (1.5) | 4 (0.8) | 10 (2.6) | 0.026 |
| Cutaneous adverse drug reactions, n (%) | 13 (1.4) | 4 (0.8) | 9 (2.3) | 0.048 |
| Visual defect, n (%) | 5 (0.5) | 2 (0.4) | 3 (0.8) | 0.655 |
| Others, n (%) | 43 (4.7) | 13 (2.4) | 30 (7.7) | <0.001 |
| Death, n (%) | 38 (4.1) | 3 (0.6) | 35 (9.0) | <0.001 |
| Medication interruption, n (%) | 83 (9) | 45 (8.5) | 38 (9.7) | 0.506 |
| LTFU, n (%) | 11 (1.2) | 5 (0.9) | 6 (1.5) | 0.542 |
| Patients experiencing an SAE, medication interruption, or LTFU, n (%) | 199 (21.6) | 88 (16.6) | 111 (28.5) | <0.001 |

Abbreviations: IQR: interquartile range; LFTU: lost to follow-up; PZA: pyrazinamide; SAE: serious adverse event
