# Supplementary material for: Impact of pyrazinamide usage on serious adverse events in elderly tuberculosis patients: A multicenter cohort study
Source: PLoS One. 2024 Sep 26;19(9):e0309902. doi: 10.1371/journal.pone.0309902 (PMC11426458; doi:10.1371/journal.pone.0309902)
Supplement: S3 Table — (DOCX) [file pone.0309902.s003.docx]

Supporting Table 3. Baseline characteristics and treatment outcomes based on the presence of severe adverse events in elderly tuberculosis patients with pyrazinamide

| Variables | Total, n = 356 | SAE,  n = 84 (23.6%) | No SEA,  n = 272 (76.4%) | *P* value |
| --- | --- | --- | --- | --- |
| Baseline characters | | | | |
| Age, years | 75.67 ± 7.21 | 76.99 ± 7.37 | 74.55 ± 6.81 | 0.005 |
| Males, n (%) | 211 (59.3) | 50 (59.5) | 161 (59.2) | 0.957 |
| BMI, kg/m^2^ | 21.97 ± 3.40 | 21.94 ± 3.04 | 21.99 ± 3.32 | 0.905 |
| Ever smoker (%) | 175 (44.9) | 11 (32.4) | 164 (46.1) | 0.124 |
| Re-treatment. n/N (%) | 59/351 (16.8) | 13/81 (16.0) | 46/270 (17.0) | 0.835 |
| Extrapulmonary TB, n (%) | 34 (9.6) | 12 (14.3) | 22 (8.1) | 0.091 |
| Multi-lobe infiltration, n/N (%) | 311/349 (89.1) | 79/83 (95.2) | 232/266 (87.2) | 0.042 |
| Cavitary lesion, n/N (%) | 44/345 (12.8) | 9/83 (10.8) | 35/262 (13.4) | 0.549 |
| AFB Smear positive, n/N (%) | 198/283 (70.0) | 49/74 (66.2) | 149/209 (71.3) | 0.413 |
| Chronic pulmonary disease, n (%) | 29 (8.1) | 10 (11.9) | 19 (7.0) | 0.150 |
| Renal disease, n (%) | 14 (3.9) | 6 (7.1) | 8 (2.9) | 0.083 |
| Liver disease, n (%) | 8 (2.2) | 0 (0.0) | 8 (2.9) | 0.112 |
| Cancer, n (%) | 47 (13.2) | 8 (9.5) | 39 (14.3) | 0.255 |
| Hemoglobin, g/dL^a^ | 12.11 ± 1.77 | 12.17 ± 5.11 | 12.34 ± 1.70 | 0.767 |
| Albumin, g/dL^b^ | 3.75 ± 0.63 | 3.51 ± 0.61 | 3.83 ± 0.62 | <0.001 |
| Total bilirubin, mg/dL ^c^ | 0.58 ± 0.28 | 0.60 ± 0.30 | 0.58 ± 0.28 | 0.658 |
| AST, IU/L ^d^ | 26.94 ± 19.97 | 29.71 ± 30.66 | 25.86 ± 12.55 | 0.280 |
| ALT, IU/L ^e^ | 19.99 ± 16.64 | 20.32 ± 21.96 | 19.82 ± 13.50 | 0.812 |
| Creatinine(mg/dL) ^f^ | 0.90 ± 0.60 | 0.99 ± 0.58 | 0.85 ± 0.54 | 0.060 |
| Treatment Outcomes | | | | |
| Treatment Success, n (%) | 320 (89.9) | 58 (69.0) | 262 (96.3) | *<0.001* |
| Medication interruption, n (%) | 34 (9.6) | 23 (27.4) | 11 (4.0) | *<0.001* |
| LTFU, n (%) | 5 (1.4) | 1 (1.2) | 4 (1.5) | 0.849 |

Abbreviations: AFB: Acid-Fast Bacillus; ALT: alanine aminotransferase; AST: aspartate aminotransferase; BMI: Body mass index; IQR: interquartile range; LFTU: lost to follow-up; PZA: pyrazinamide; SAE: serious adverse event; TB: Tuberculosis

^a^ Total n = 326; SAE n = 79; No SAE n = 247

^b^ Total n = 313; SAE n = 78; No SAE n = 235

^c^ Total n = 312; SAE n = 78; No SAE n = 234

^d^ Total n = 322; SAE n = 79; No SAE n = 243

^e^ Total n = 322; SAE n = 79; No SAE n = 243

^f^ Total n = 324; SAE n = 79; No SAE n = 245
