# Supplementary material for: Impact of pyrazinamide usage on serious adverse events in elderly tuberculosis patients: A multicenter cohort study
Source: PLoS One. 2024 Sep 26;19(9):e0309902. doi: 10.1371/journal.pone.0309902 (PMC11426458; doi:10.1371/journal.pone.0309902)
Supplement: Attachment — Submitted filename: Response to reviewers comments.docx [file pone.0309902.s004.docx]

**Journal requirements:**

When submitting your revision, we need you to address these additional requirements.

1. Please ensure that your manuscript meets PLOS ONE's style requirements, including those for file naming. The PLOS ONE style templates can be found at

https://journals.plos.org/plosone/s/file?id=wjVg/PLOSOne_formatting_sample_main_body.pdf and

<https://journals.plos.org/plosone/s/file?id=ba62/PLOSOne_formatting_sample_title_authors_affiliations.pdf>

- We checked style requirements.

2. Thank you for stating the following financial disclosure:

[his study was supported by a grant (BCRI24033) from Chonnam National University Hospital Biomedical Research Institute. ].

Please state what role the funders took in the study. If the funders had no role, please state: "The funders had no role in study design, data collection and analysis, decision to publish, or preparation of the manuscript."

If this statement is not correct you must amend it as needed.

Please include this amended Role of Funder statement in your cover letter; we will change the online submission form on your behalf.

- We added funders and stated the role of the funders.

3. We note that you have indicated that there are restrictions to data sharing for this study. For studies involving human research participant data or other sensitive data, we encourage authors to share de-identified or anonymized data. However, when data cannot be publicly shared for ethical reasons, we allow authors to make their data sets available upon request. For information on unacceptable data access restrictions, please see <http://journals.plos.org/plosone/s/data-availability#loc-unacceptable-data-access-restrictions>.

Before we proceed with your manuscript, please address the following prompts:

a) If there are ethical or legal restrictions on sharing a de-identified data set, please explain them in detail (e.g., data contain potentially identifying or sensitive patient information, data are owned by a third-party organization, etc.) and who has imposed them (e.g., a Research Ethics Committee or Institutional Review Board, etc.). Please also provide contact information for a data access committee, ethics committee, or other institutional body to which data requests may be sent.

b) If there are no restrictions, please upload the minimal anonymized data set necessary to replicate your study findings to a stable, public repository and provide us with the relevant URLs, DOIs, or accession numbers. Please see http://www.bmj.com/content/340/bmj.c181.long for guidelines on how to de-identify and prepare clinical data for publication. For a list of recommended repositories, please see https://journals.plos.org/plosone/s/recommended-repositories. You also have the option of uploading the data as Supporting Information files, but we would recommend depositing data directly to a data repository if possible**.**

- We specifically outlined the data sharing policy of this study.

“The data that support the findings of this study are available from Korea Disease Control and Prevention Agency, however, restrictions apply. Data will be provided by the Korea Disease Control and Prevention Agency (Tae-hyoun Kim, D.V.M., Ph.D. whitevet81@korea.kr, Division of Bacterial Disease Research, Center for Infectious Disease Research, National Institute of Health, Korea Disease Control and Prevention Agency, Cheongju, South Korea) upon reasonable request..”

**Reviewer #1:**

Reviewer #1: This is a retrospective observational study specifically in an important population group (the elderly) which is often neglected in clinical research. The study emphasizes the importance and benefits of using pyrazinamide regardless of age in treatment success for TB. It adds to the body of evidence to help guide treatment of TB in the elderly.

- Thank you for your helpful comments.

I have some comments and suggestions for the authors to address.

Line 120-121: please state the incidence of TB in the Republic of Korea and also among the elderly population.

- We added it as you recommended (Line 119).

Table 1: a. What does overt smoker mean? Does overt smoker mean current smoker?

- Thank you for your valuable comment. In our paper, the term 'overt smoker' includes both current and ex-smokers. To avoid confusion, we have replaced 'overt smoker' with 'ever smoker'.

b. Does re-treatment mean that the patient had been previously treated for TB? This seems to be a high proportion of patients. Please explain.

- We understand that your definition of re-treatment aligns with ours. Re-treatment cases include patients who have previously undergone TB treatment, encompassing those with recurrence after treatment and treatment failure and interruptions during prior treatments.

Based on the nationwide data from the annual report on notified tuberculosis patients in Korea for 2023 by the Korea Disease Control and Prevention Agency (available at reference 21), re-treatment cases accounted for 13.3% (2,565 out of 19,540 patients) of all TB patients in South Korea. Considering the age of the enrolled patients in this study (elderly, age ≥ 65), we believe that the rate of re-treatment cases was not high compared to that in the nationwide data.

Table 2: Some of the terminology of SAEs are a little unusual. For example, with general weakness, do you mean generalized weakness? Please find alternatives for “gastrointestinal trouble”, “cutaneous trouble”.

- Thank you for your valuable comment. We changed terms to generalized weakness, gastrointestinal adverse drug reactions, and cutaneous adverse drug reactions.

Table 3: Suggest 2 decimal places for values. As chronic pulmonary disease has the highest OR in multivariate regression, you may need to explain the definition of chronic pulmonary disease.

For AFB-positive, is this only for pulmonary TB, or is extra-pulmonary TB smear included in this category?

- Thank you for your valuable comments. We adjusted the values to 2 decimal places in Table 3.

The definition of chronic pulmonary disease in this study includes patients with asthma, chronic bronchitis, emphysema, and other chronic lung disease who have ongoing symptoms such as dyspnea or cough, with mild or moderate activity. We added it in the Methods section (Line 172).

For AFB-positive, this is only for pulmonary TB and we added it in the Methods section (Line 172)

Line 262: Was the duration of HREZ shorter than HRE because recommended treatment for HRE is 9 months compared with 6 months for HREZ? A shorter duration of treatment indicating a better outcome is a very generalized statement as other factors need to be considered such as treatment success, similar or reduced adverse events etc. Please rephrase.

- Thank you for your valuable comments. We rephrase the sentence to “Considering treatment duration, the PZA-containing regimen (HREZ) is much shorter than the regimen without PZA (HRE), with durations of 6 months versus 9 months, respectively. Therefore, patients receiving PZA had a shorter treatment duration, which could result in less adverse drug reactions, medication interruption, and instances of LTFU, leading to better outcomes.” (Line 280)

Line 271: Could it be that the group not prescribed PYZ had poorer outcomes because they had poorer health in general? You implied this in line 274-275. 9 of 34 patients died in the “without PYZ” group and 11 of 34 had “general weakness” which appeared to be the main causes of SAEs.

- Thank you for your valuable comments. We agreed with your comment that patients without PZA might have poorer health condition. However, due to retrospective design of this study, we did not know the exact health conditions of enrolled patients which could be a limitation of this study. We added it in the Discussion section (Line 296)

Line 278: Your definition of death was all-cause mortality and not specifically TB-related death. For example, a patient may die with TB, not of TB. Also, given that the “without PZA” group was 4 years older than the “with PZA” group, this already puts them at a higher risk of death. Is there any data on the causes of death in your study?

- Thank you for your valuable comments. We agreed that TB-related death would be a better outcome measurement for this study. Unfortunately, we do not have data about causes of death in enrolled patients. We added it in the limitation of this study.

Line 288: You mention presence of COPD but previously had written “chronic pulmonary disease”. Please clarify.

- Thank you for your valuable comment. We change it to chronic pulmonary disease.

Line 292-293; line 295-296: When you refer to anemia and COPD being risk factors for death, does it refer to increased mortality risk specifically in TB disease?

- Anemia and COPD are indeed risk factors for death in TB patients, as shown in our reference papers. To clarify this, we have added "death in TB patients" to our manuscript.

It would be nice to see an analysis of the “with PYZ” group divided into those who had SAE vs those who did not have SAEs, given that the other primary outcomes of LTFU and treatment interruption does not seem to have had much overall impact. This would potentially be useful in finding out risk factors for SAEs specifically within the elderly population.

- Thank you for your valuable comments. As you recommended, we analyzed patients with PZA based on the presence of SAEs. In terms of baseline characteristics, patients with SAEs were older, had more multi-lobe infiltration, and had lower serum albumin levels. Regarding treatment outcomes, patients with SAEs had a lower treatment success rate and a higher medication interruption rate. We have included these findings in the results section (line 234) and in Supporting Table 3.

Supporting Table 3. Baseline characteristics and treatment outcomes based on the presence of severe adverse events in elderly tuberculosis patients with pyrazinamide

| Variables | Total, n = 356 | SAE,  n = 84 (23.6%) | No SEA,  n = 272 (76.4%) | *P* value |
| --- | --- | --- | --- | --- |
| Baseline characters | | | | |
| Age, years | 75.67 ± 7.21 | 76.99 ± 7.37 | 74.55 ± 6.81 | 0.005 |
| Males, n (%) | 211 (59.3) | 50 (59.5) | 161 (59.2) | 0.957 |
| BMI, kg/m^2^ | 21.97 ± 3.40 | 21.94 ± 3.04 | 21.99 ± 3.32 | 0.905 |
| Ever smoker (%) | 175 (44.9) | 11 (32.4) | 164 (46.1) | 0.124 |
| Re-treatment. n/N (%) | 59/351 (16.8) | 13/81 (16.0) | 46/270 (17.0) | 0.835 |
| Extrapulmonary TB, n (%) | 34 (9.6) | 12 (14.3) | 22 (8.1) | 0.091 |
| Multi-lobe infiltration, n/N (%) | 311/349 (89.1) | 79/83 (95.2) | 232/266 (87.2) | 0.042 |
| Cavitary lesion, n/N (%) | 44/345 (12.8) | 9/83 (10.8) | 35/262 (13.4) | 0.549 |
| AFB Smear positive, n/N (%) | 198/283 (70.0) | 49/74 (66.2) | 149/209 (71.3) | 0.413 |
| Chronic pulmonary disease, n (%) | 29 (8.1) | 10 (11.9) | 19 (7.0) | 0.150 |
| Renal disease, n (%) | 14 (3.9) | 6 (7.1) | 8 (2.9) | 0.083 |
| Liver disease, n (%) | 8 (2.2) | 0 (0.0) | 8 (2.9) | 0.112 |
| Cancer, n (%) | 47 (13.2) | 8 (9.5) | 39 (14.3) | 0.255 |
| Hemoglobin, g/dL^a^ | 12.11 ± 1.77 | 12.17 ± 5.11 | 12.34 ± 1.70 | 0.767 |
| Albumin, g/dL^b^ | 3.75 ± 0.63 | 3.51 ± 0.61 | 3.83 ± 0.62 | <0.001 |
| Total bilirubin, mg/dL ^c^ | 0.58 ± 0.28 | 0.60 ± 0.30 | 0.58 ± 0.28 | 0.658 |
| AST, IU/L ^d^ | 26.94 ± 19.97 | 29.71 ± 30.66 | 25.86 ± 12.55 | 0.280 |
| ALT, IU/L ^e^ | 19.99 ± 16.64 | 20.32 ± 21.96 | 19.82 ± 13.50 | 0.812 |
| Creatinine(mg/dL) ^f^ | 0.90 ± 0.60 | 0.99 ± 0.58 | 0.85 ± 0.54 | 0.060 |
| Treatment Outcomes | | | | |
| Treatment Success, n (%) | 320 (89.9) | 58 (69.0) | 262 (96.3) | *<0.001* |
| Medication interruption, n (%) | 34 (9.6) | 23 (27.4) | 11 (4.0) | *<0.001* |
| LTFU, n (%) | 5 (1.4) | 1 (1.2) | 4 (1.5) | 0.849 |

Abbreviations: AFB: Acid-Fast Bacillus; ALT: alanine aminotransferase; AST: aspartate aminotransferase; BMI: Body mass index; IQR: interquartile range; LFTU: lost to follow-up; PZA: pyrazinamide; SAE: serious adverse event; TB: Tuberculosis

^a^ Total n = 326; SAE n = 79; No SAE n = 247

^b^ Total n = 313; SAE n = 78; No SAE n = 235

^c^ Total n = 312; SAE n = 78; No SAE n = 234

^d^ Total n = 322; SAE n = 79; No SAE n = 243

^e^ Total n = 322; SAE n = 79; No SAE n = 243

^f^ Total n = 324; SAE n = 79; No SAE n = 245

Minor comments:

Line 74: Republic of Korea

Line 76: loss to follow-up

Line 102: more vulnerable

Line 166: Change cavity lesion to cavitary lesion in keeping with Table 1

- Thank you for your detailed comments. We changed these as you recommended.

Reviewer #2: 1. The younger group (<65 years) shows lower risk of liver disfunction in comparison to >65 years old group as in the supplementary table 1. Does this valid both for HRZE group and HRE group? If this is valid only for HRZE group, it may be the reason to avoid PZA for the elderly.

- We appreciate your detailed review comments. As shown in Table 2, there was no difference in hepatotoxicity between elderly TB patients receiving PZA and those not receiving PZA. Further statistical analysis confirmed that there were no differences in hepatotoxicity between the younger group or across all age groups.

Hepatotoxicity in all TB patients based on PZA usage

| Variables | Total,  n = 921 | Without PZA,  n = 48 (5.2%) | With PZA,  n = 873 (94.8%) | *P* value |
| --- | --- | --- | --- | --- |
| Hepatotoxicity, n (%) | 47 (5.1) | 4 (8.3) | 43 (4.9) | 0.301 |

Hepatotoxicity in younger TB patients (<65 years) based on PZA usage

| Variables | Total,  n = 531 | Without PZA,  n = 14 (2.6%) | With PZA,  n = 517 (97.4%) | *P* value |
| --- | --- | --- | --- | --- |
| Hepatotoxicity, n (%) | 17 (3.2) | 0 (0.0) | 17 (3.3) | 1.000 |

2. Did you analyse for >75 years old group only for the risk factors of ADR? US guidelines says "Consequently, some experts avoid the use of PZA during the intensive phase among patients >75 years of age. "

- Thank you for your valuable comment. We analyzed our data as you recommended. Very old age group also showed same as elderly group in this study. We added it in the Result section (line 226) and supporting table 1.

Supporting Table 1. Baseline characteristics and treatment results based on pyrazinamide usage in patients aged 75 years and older

| Variables | Total, n = 199 | Without PZA,  n = 26 (13.1%) | With PZA,  n = 173 (86.9%) | *P* value |
| --- | --- | --- | --- | --- |
| Baseline characters | | | | |
| Age, years | 81.62 ± 4.64 | 82.54 ± 4.58 | 81.14 ± 4.61 | 0.150 |
| Males, n (%) | 89 (44.7) | 10 (38.5) | 79 (45.7) | 0.491 |
| BMI, kg/m^2^ | 21.84 ± 3.61 | 22.61 ± 4.37 | 21.74 ± 3.37 | 0.366 |
| Ever smoker (%) | 62 (31.2) | 8 (30.8) | 54 (31.2) | 0.964 |
| Re-treatment. n/N (%) | 28/197 (14.2) | 4/25 (16.0) | 24/172 (14.0) | 0.784 |
| Extrapulmonary TB, n (%) | 24 (12.1) | 5 (19.2) | 19 (11.0) | 0.229 |
| Multi-lobe infiltration, n/N (%) | 183/198 (92.4) | 26/26 (100.0) | 157/172 (91.3) | 0.117 |
| Cavitary lesion, n/N (%) | 17/196 (8.7) | 1/26 (3.8) | 16/170 (9.4) | 0.348 |
| AFB Smear positive, n/N (%) | 111/167 (66.5) | 12/18 (66.7) | 99/149 (66.4) | 0.985 |
| Chronic pulmonary disease, n (%) | 22 (11.1) | 6 (23.1) | 16 (9.2) | 0.036 |
| Renal disease, n (%) | 16 (8.0) | 4 (15.4) | 12 (6.9) | 0.140 |
| Liver disease, n (%) | 3 (1.5) | 1 (3.8) | 2 (1.2) | 0.344 |
| Cancer, n (%) | 21 (10.6) | 3 (11.5) | 18 (10.4) | 0.861 |
| Hemoglobin, g/dL^a^ | 11.57 ± 1.52 | 11.45 ± 1.62 | 11.53 ± 1.51 | 0.802 |
| Albumin, g/dL^b^ | 3.57 ± 0.63 | 3.15 ± 0.71 | 3.62 ± 0.59 | 0.001 |
| Total bilirubin, mg/dL ^c^ | 0.65 ± 0.61 | 1.11 ± 1.36 | 0.58 ± 0.29 | 0.069 |
| AST, IU/L ^d^ | 20.26 ± 20.52 | 46.08 ± 55.71 | 27.78 ± 23.51 | 0.125 |
| ALT, IU/L ^e^ | 20.26 ± 20.52 | 24.13 ± 27.61 | 18.94 ± 18.16 | 0.230 |
| Creatinine(mg/dL) ^f^ | 0.96 ± 0.50 | 1.09 ± 0.52 | 0.93 ± 0.48 | 0.147 |
| Treatment Results | | | | |
| Treatment Success, n (%) | 165 (82.9) | 16 (61.5) | 149 (86.1) | *0.002* |
| SAEs, n (%) | 62 (31.2) | 11 (42.3) | 51 (29.5) | 0.188 |
| Time to first SAE, median days (IQR) | 35 (13-89) | 28 (13-63) | 46 (13-91) | 0.693 |
| Treatment duration, median days (IQR) | 185 (179-217) | 206 (20-180) | 185 (180-203) | 0.273 |
| Medication interruption, n (%) | 20 (10.1) | 2 (7.7) | 18 (10.4) | *0.668* |
| LTFU, n (%) | 5 (2.5) | 2 (7.7) | 3 (1.7) | 0.128 |

Abbreviations: AFB: Acid-Fast Bacillus; ALT: alanine aminotransferase; AST: aspartate aminotransferase; BMI: Body mass index; IQR: interquartile range; LFTU: lost to follow-up; PZA: pyrazinamide; SAE: serious adverse event; TB: Tuberculosis

^a^ Total n = 182; without PZA n = 24; with PZA n = 158

^b^ Total n = 178; without PZA n = 24; with PZA n = 154

^c^ Total n = 179; without PZA n = 24; with PZA n = 155

^d^ Total n = 181; without PZA n = 24; with PZA n = 157

^e^ Total n = 181; without PZA n = 24; with PZA n = 157

^f^ Total n = 181; without PZA n = 24; with PZA n = 157
